# Supplementary material for: Brucella abortus Strain RB51 Administered to Prepubescent Water Buffaloes, from Vaccination to Lactation: Kinetics of Antibody Response and Vaccine Safety
Source: Microorganisms. 2023 Aug 13;11(8):2078. doi: 10.3390/microorganisms11082078 (PMC10459664; doi:10.3390/microorganisms11082078)
Supplement: Supplementary file 1 [file microorganisms-11-02078-s001.zip › Table S1 RB51_rev.FS_12-08-2023.pdf]

Table S1. Non-parametric Mann Whitney test for RB51-CFT antibody titers**.** Comparison of logaritmic values of RB51-CFT between vaccinated and control animals for each time point. (p value, red cells $p < 0.05$).

**Weeks post vaccination**	**mean_Controls**	**Mean_Vaccinated**	**U**	***p*-value**
0	0	0	90	#N/D
1	0	18.631	177	0
2	0	22.849	174	0
3	0	31.396	171	0.001
4	0.471	20.162	169	0.001
5	0	20.763	168	0.001
6	0	17.999	174	0
7	0	12.089	159	0.003
8	0	13.099	168	0.001
10	0	9.078	159	0.003
11	0	5.714	144	0.015
12	0	4.602	153	0.005
13	0	5.644	153	0.006
14	0	4.801	150	0.008
15	0	4.673	150	0.008
16	0	3.164	129	0.056
17	0	1.998	126	0.072
18	0	2.490	132	0.045
19	0	1.635	123	0.091
20	0	2.102	132	0.045
21	0	16.975	180	0
22	0	18.815	180	0
23	0	32.126	180	0
24	0	25.412	174	0
25	0	25.484	174	0

26	0	24.580	174	0
27	0	21.535	171	0
28	0	23.429	168	0
29	0	16.876	168	0
30	0	16.074	168	0
31	0	14.059	168	0
32	0	13.150	165	0
33	0	11.386	168	0
34	0	13.843	168	0
35	0	12.452	168	0
36	0	11.201	168	0
37	0	13.952	140	0
40	0	8.054	137.5	0.001
41	0	5.251	120	0.01
42	0	6.774	132.5	0.002
43	0	8.318	130	0.002
44	0	6.619	132.5	0.002
45	0	6.753	132.,5	0.001
46	0	6.854	130	0.002
48	0	4.862	120	0.01
50	0	5.853	130	0.002
52	0	6.243	132.5	0.002
54	0	4.782	130	0.002
56	0	4.844	130	0.002
59	0	1.532	80	0.324
61	0	3.603	115	0.011
63	0	6.408	127.5	0.002
67	0	5.312	127.5	0.002
71	0	2.837	112.5	0.015
75	0	1.654	92.5	0.121
80	0	1.774	87.5	0.183

84	0	2.493	102.5	0.048
93	0	1.168	80	0.324
97	0	1.631	95	0.097
101	0	1.356	87.5	0.183
105	0	1.509	90	0.15
109	0	0.973	90	0.149
