# Supplementary figures and images for: Brucella abortus Strain RB51 Administered to Prepubescent Water Buffaloes, from Vaccination to Lactation: Kinetics of Antibody Response and Vaccine Safety
Source: Microorganisms. 2023 Aug 13;11(8):2078. doi: 10.3390/microorganisms11082078 (PMC10459664; doi:10.3390/microorganisms11082078)

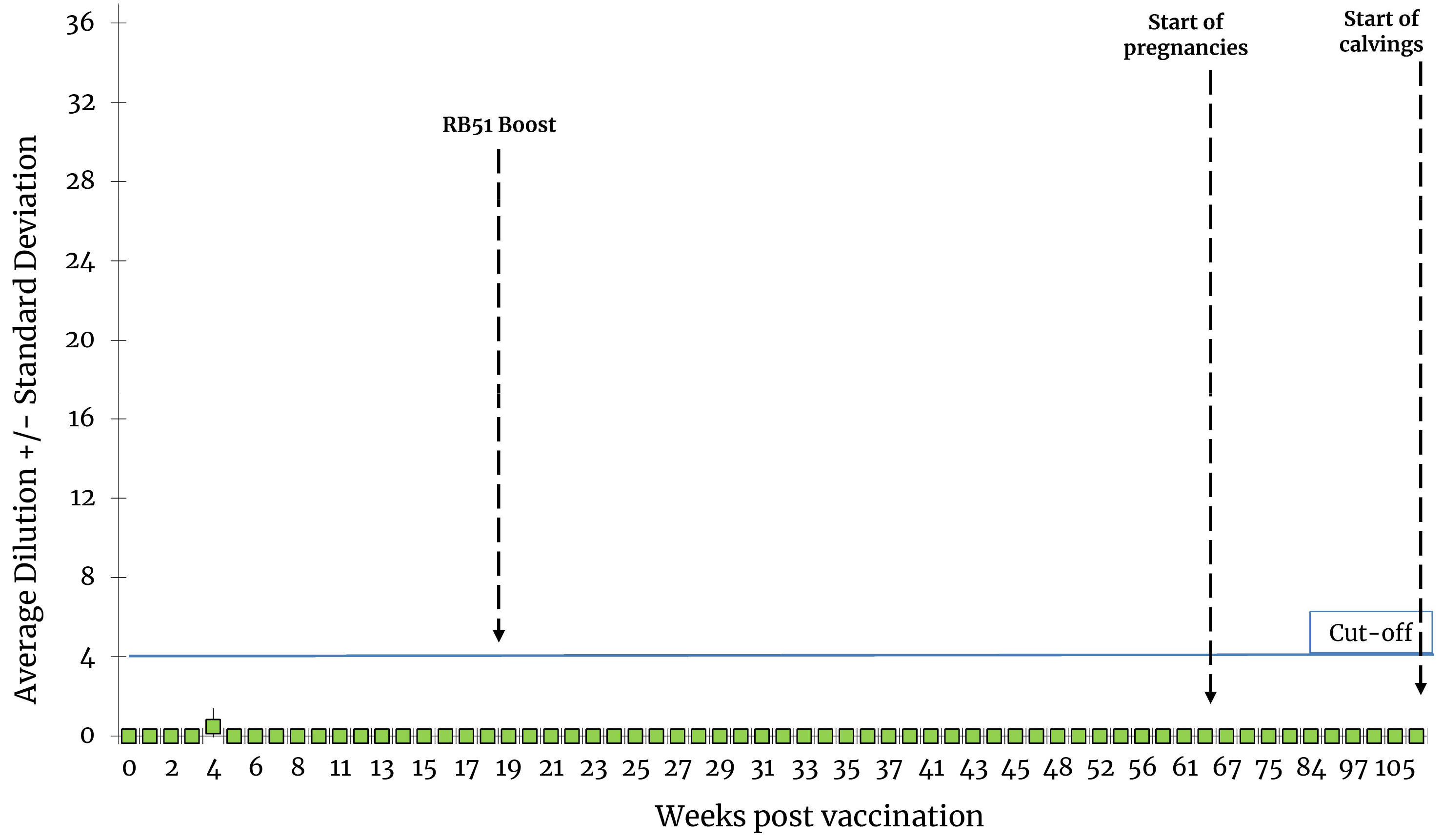

Supplement: Supplementary file 1 [file microorganisms-11-02078-s001.zip › figS1.jpg]

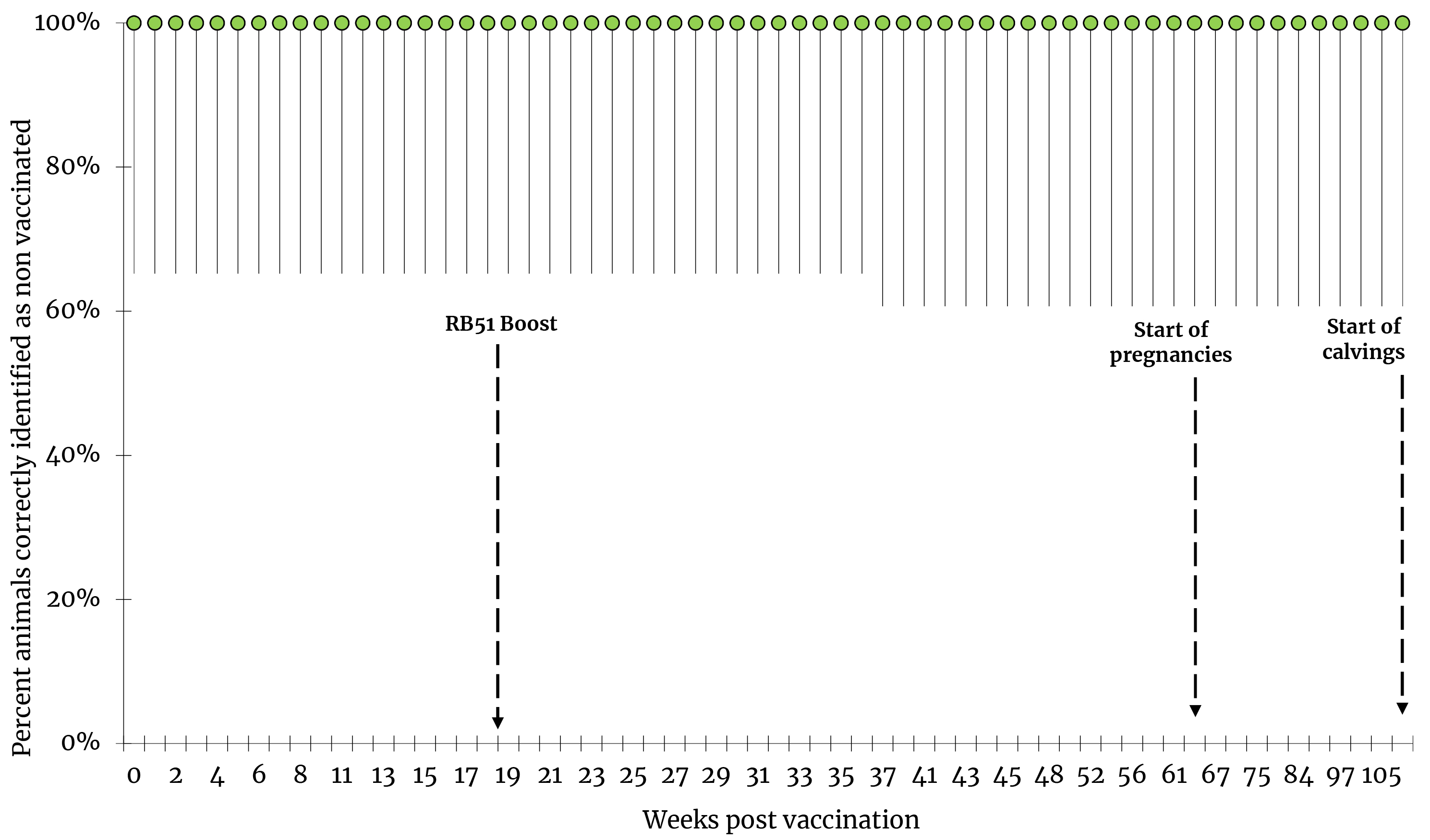

Supplement: Supplementary file 1 [file microorganisms-11-02078-s001.zip › figS2.jpg]
